# Phenology of the Diamondback Moth (*Plutella xylostella*) in the UK and Provision of Decision Support for *Brassica* Growers

**DOI:** 10.3390/insects11020118

**Published:** 2020-02-11

**Authors:** Charlotte Wainwright, Sascha Jenkins, Daniel Wilson, Marian Elliott, Andrew Jukes, Rosemary Collier

**Affiliations:** 1 Rothamsted Research, Department of Computational and Analytical Sciences, West Common, Harpenden, Hertfordshire AL5 2JQ, UK; cwainwri@nd.edu; 2 Warwick Crop Centre, School of Life Sciences, Wellesbourne Campus, University of Warwick, Wellesbourne, Warwick CV35 9EF, UK; sascha.jenkins@warwick.ac.uk (S.J.); daniel.wilson@warwick.ac.uk (D.W.); marian.elliott@warwick.ac.uk (M.E.); andrew.jukes@warwick.ac.uk (A.J.)

**Keywords:** *Plutella xylostella*, pheromone trap, citizen science, phenology, *Brassica* crop, migration, monitoring, decision support, migrant moths

## Abstract

In the UK, severe infestations by *Plutella xylostella* occur sporadically and are due mainly to the immigration of moths. The aim of this study was to develop a more detailed understanding of the phenology of *P. xylostella* in the UK and investigate methods of monitoring moth activity, with the aim of providing warnings to growers. *Plutella xylostella* was monitored using pheromone traps, by counting immature stages on plants, and by accessing citizen science data (records of sightings of moths) from websites and Twitter. The likely origin of migrant moths was investigated by analysing historical weather data. The study confirmed that *P. xylostella* is a sporadic but important pest, and that very large numbers of moths can arrive suddenly, most often in early summer. Their immediate sources are countries in the western part of continental Europe. A network of pheromone traps, each containing a small camera sending images to a website, to monitor *P. xylostella* remotely provided accessible and timely information, but the particular system tested did not appear to catch many moths. In another approach, sightings by citizen scientists were summarised on a web page. These were accessed regularly by growers and, at present, this approach appears to be the most effective way of providing timely warnings.

## 1. Introduction

The diamondback moth, *Plutella xylostella* L. (Lepidoptera: Plutellidae), is an important pest of brassicaceous crops (e.g., cabbage, broccoli, and cauliflower) worldwide [1,2]. Direct damage occurs as a result of larval feeding, which can have significant impacts on crop quality and, in the case of severe infestations, plant survival. The relatively short life-cycle of *P. xylostella* [3] means that it can complete several generations in a year in most locations. This is particularly important in tropical regions, where it is a pernicious pest and may complete up to 14 generations in a year [4].

Following wide-scale application of insecticides to *Brassica* crops over recent decades [5], *P. xylostella* has developed high levels of resistance to a number of chemical and biological insecticides, particularly in tropical regions [6]. This presents a considerable challenge for growers in many parts of the world and costs the global agricultural industry an estimated US $4–5 billion in pest control costs and crop losses annually [5].

In the UK and other northern European countries, severe infestations by *P. xylostella* occur sporadically. When *P. xylostella* infestations do occur, they can be extremely destructive and may sometimes catch growers unawares, due to the small size of the eggs and first instar larvae; the latter mine into the spongy mesophyll tissue [1] and are therefore inconspicuous. It is believed that *P. xylostella* does not overwinter very successfully in the UK [7] and other temperate locations due to the lack of a defined, cold-resilient overwintering stage [1]. Zalucki and Furlong [8] suggest that it is at the edge of its range in the southern UK (using a CLIMEX model and weather data from Oxford for 1853–2010). *Plutella xylostella* is known to be highly migratory [7,9] and most major infestations in the UK appear to be due to the progeny of immigrant moths and the subsequent generations they produce during the summer; this conclusion is supported also by the modelling work of Zalucki and Furlong [8]. However, recent observations by a *Brassica* grower in Somerset, UK, and confirmed by one of the authors (R. Collier), suggest that in mild winters it may be possible for this species to overwinter (larvae were found in mid-January 2018). In the future, increasing temperatures as a result of climate change may alter the lifecycle and phenology of this pest in temperate regions; it may arrive earlier in the year and larger numbers may overwinter [10].

For many of the pest insects of agricultural crops that overwinter in the UK it is possible to develop weather-based forecasting systems to predict when activities such as oviposition will occur, using accumulated temperatures from the putative end of diapause in late winter/early spring [11]. In the case of immigrant *P. xylostella* this is not possible, because the previous whereabouts of migrant populations, and the environmental conditions they have been exposed to, are usually unknown. The aim of the research described here was to develop a more detailed understanding of the phenology of *P. xylostella* in the UK and of methods of monitoring immigration to provide improved advice to growers to help them to react to possible infestations by this pest.

## 2. Materials and Methods

### 2.1. Phenology of P. xylostella in the UK

Adult male *P. xylostella* were monitored using Delta pheromone traps (Agralan Ltd., Swindon, UK; pheromone is a blend of (Z)-11-Hexadecenal, (Z)-11-Hexadecenyl acetate, (Z)-11-Hexadecen-1-ol). The traps were secured just above crop height in commercial crops or experimental plots of *Brassica* vegetables. In some cases, the immature stages were monitored by examining individual insecticide-free *Brassica* plants (Brussels sprout) for eggs, larvae, and pupae. All the foliage on each of a pre-determined number of plants (usually 20) was searched for insects. There are two main sets of data: (1) data on all life stages for 5 sites in South Lincolnshire, UK, during 1996–2000 (close to the villages of Butterwick, Donington, Friskney, Holbeach (all commercial crops), and Kirton (experimental farm), respectively) and (2) adult male moths were monitored using pheromone traps at Wellesbourne in Warwickshire from 1996 to 2019. The sources of the data and periods of data collection are indicated with the Results.

Additionally, in both 2015 and 2016, a network of 10 pheromone traps was established in England and Scotland to monitor *P. xylostella*. The overall aim was to determine whether running such a network of traps would be viable and informative to growers. The traps were supplied by Trapview (www.trapview.com) and were hosted by growers of *Brassica* crops. The locations of the traps were as follows: Warwickshire (1 trap at Warwick Crop Centre, Wellesbourne), Devon (1 site), Somerset (1 site), Kent (2 sites), Essex (1 site), Lincolnshire (1 site), Yorkshire (1 site), and Perthshire (2 sites). Traps were located within fields of *Brassica* crops which were either swede, cauliflower, Brussels sprout, cabbage, or salad brassicas. Each trap contained a pheromone lure (Sentomol Limited; blend of (Z)-11-Hexadecenal, (Z)-11-Hexadecenyl acetate, (Z)-11-Hexadecen-1-ol), a sticky base to capture the moths, and a small camera that photographed the sticky base once each day. The camera was powered by a solar cell. The image was downloaded onto the website managed by Trapview and the images of the captures by all the traps were visible to all the trap hosts. “Conventional” pheromone traps were run in parallel to the “Trapview traps” at 3 of the sites although the original intention was to run them at all sites. The lures in all traps were replaced at the recommended intervals and the sticky bases were replaced as and when necessary. The data from the Trapview traps were downloaded from the Trapview site and checked and corrected using the images. Data from the other traps were sent to Warwick Crop Centre at the end of the season.

### 2.2. Information from Databases and Twitter

In northern Europe, sightings of adult *P. xylostella* are recorded on national observation portals in Belgium (https://waarnemingen.be/), The Netherlands (https://waarneming.nl/), Sweden (http://www.artportalen.se/), Norway (http://www.artsobservasjoner.no/), Finland (https://laji.fi/), and the UK (http://www.atropos.info/site/). These are websites on which naturalists/citizen scientists are able to upload information on species that they have seen, complete with time and location data, and often with photographs. The data are updated continuously and are easily accessible to others. In the UK, the Atropos website (http://www.atropos.info/site/) is focused specifically on migrant Lepidoptera. Sightings of *P. xylostella* and other species of moth are also reported on Twitter and there is at least one Twitter account that focuses specifically on migrant species in the UK (@MigrantMothUK). Much of the information they report is based on captures in light traps (the type of light trap used will vary). Data on *P. xylostella* were accessed from all these sources for a number of years. Information was generally sourced for a full year (1 January–31 December). The exceptions were for data for the UK from Twitter and the Atropos website in 2017 (24 May–30 October), 2018 (4 April–1 November), and 2019 (15 April–31 December).

### 2.3. Identifying the Origin of Migrant Moths in the UK

Accounts from observers, growers, and light trap operators on social media indicated movements of extremely high numbers of *P. xylostella* into the UK around 1 June 2016. To visualise the initial spread of *P. xylostella* from its overwintering areas to higher latitudes across northern Europe, data from the citizen science observation portals was used.

The *P. xylostella* records from the Rothamsted Insect Survey light trap network in the UK and the citizen science observations across northern Europe were used to study the movement across the continent and the conditions preceding the initial mass migration into the UK on 1 June 2016. Using the NCEP reanalysis temperature and wind fields [12], a video was produced that overlaid the citizen science records on the meteorological data to illustrate the effects of the wind conditions on *P. xylostella* movement. The video clearly showed that large-scale movements of *P. xylostella* are mainly based on the prevailing wind conditions, with higher temperatures also an indication that mass movement is likely to occur.

To test whether it was possible to identify the source location for the initial outbreak incursion of *P. xylostella* into the UK on 1 June, the NOAA HYSPLIT READY model [13,14] (available online at https://ready.arl.noaa.gov) was used. The model was used in frequency mode, which initializes a new trajectory from a single starting location every 3 h for a user-defined number of days. This enables illustration of mass movement over certain time periods, and is helpful for instances when the flight limitations of an insect species or their preferential take-off conditions may be unknown. For the trajectory end-point, the location of a light trap in Kimpton, Hertfordshire, was used, which recorded its first 2016 instance of *P. xylostella* on 1 June, with 16 moths caught in the light trap on that night. Since it was unknown whether there is a delay between the moths’ initial arrival in the UK and the appearance of the moths in light traps and in a field setting, trajectories were initialized every 3 h from 07:00 on 4 June backwards to 10:00 on 30 May. The meteorological data used in the trajectory analysis are from the NCEP/NCAR reanalysis dataset. A flight height of 500 m was used for the trajectory analysis, with a flight duration of 24 h. Since the true flight duration and minimum survivable temperature or pressure for *P. xylostella* have yet to be determined, it is also possible that higher flight heights may be utilised, which would allow the moths to travel even further in a given time period, due to the general increase in wind speed with height.

### 2.4. Grower Experience of the Influx of P. xylostella into the UK in 2016

A workshop for growers was held in late January 2017, organised by the UK Agriculture and Horticulture Development Board (AHDB) [15], and included breakout groups to ascertain the level of damage in different *Brassica* crops in 2016 and the management approaches used. In addition, the link to a short online questionnaire was circulated to growers to determine whether they used pheromone traps and when they first observed moths, eggs, and larvae in their crops.

### 2.5. Using Citizen Science and Grower Monitoring Data to Provide Warnings for UK Growers

Following the workshop, a web page was set up each year on the University of Warwick website to provide daily summaries of the observations posted on the European portals and through Twitter in the UK with the aim of providing UK growers with as much warning as possible of large influxes of *P. xylostella* and *Autographa gamma* (silver Y moth), which is mainly a pest of lettuce crops [16]. Where approximations of numbers seen were given (mainly on Twitter) a “conservative” estimate was used. This activity was funded by the AHDB during 2017–2019. The web page was supported by weekly email “bulletins” on pest activity sent to interested growers by the AHDB and, in the case of large influxes of moths, additional email alerts were sent to *Brassica* growers. Additionally, in 2018 and 2019, a small group of growers/agronomists set up a network of “conventional” pheromone traps to monitor *P. xylostella* [16]. These were located from Devon in south-west England to central Scotland in the north. Trap operators forwarded information to Rosemary Collier and the information was summarised on a second web page. It was up to the trap operators to decide how many traps to use and where to site them.

## 3. Results

### 3.1. Phenology of P. xylostella in the UK

Table 1 summarises data from five locations in Lincolnshire in terms of the abundance of adult male *P. xylostella* captured in pheromone traps, the numbers of larvae found on 20 Brussels sprout plants, and the timings of peaks in moth and larval abundance. Adult males were most abundant in 1996. Peak numbers of male moths were captured between late June and mid-August and, with the exception of 1999, peak numbers of larvae were seen 6–17 days later.

**Figure 1:**
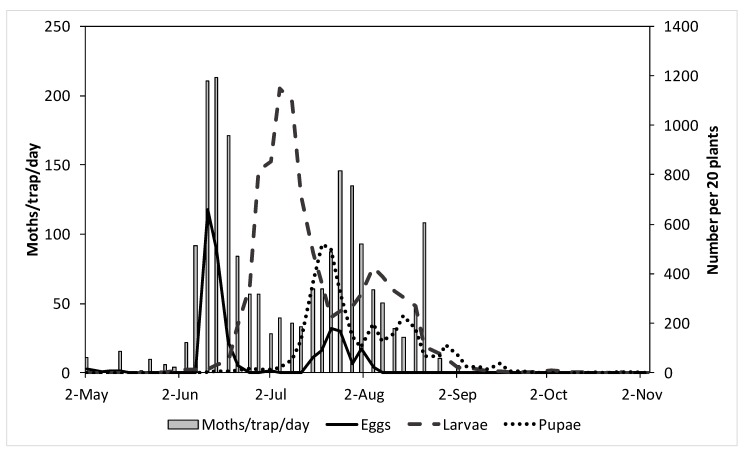
Numbers of adult male *P. xylostella* per trap per day (axis on left) and eggs, larvae, and pupae per 20 plants (axis on right) at five sites in Lincolnshire in 1996.

Figure 1 shows the numbers of male moths captured in pheromone traps (moths/trap/day) and the numbers of eggs and larvae found on 20 Brussels sprout plants at the five sites in Lincolnshire in 1996, the year when *P. xylostella* was most abundant. These data confirm that when male moths were captured in traps, female moths were laying eggs on host plants. The graph strongly suggests that the second peak in numbers of moths resulted from the immature stages developing in the plots and surrounding crops rather than a new influx of migrant moths.

Table 2 summarises moth captures in pheromone traps at Wellesbourne, Warwickshire, between 2006 and 2019 (June–September inclusive). The total numbers captured ranged from 1 to 440 per trap. The most significant infestation was in 2016. Peak numbers of moths were usually captured in June or July.

**Figure 2:**
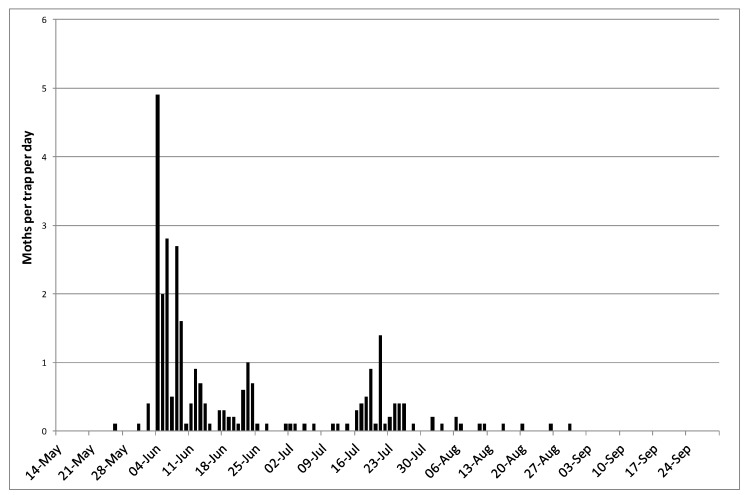
Mean numbers of male *Plutella xylostella* captured at 10 locations in the UK in 2016 using Trapview traps.

**Figure 3:**
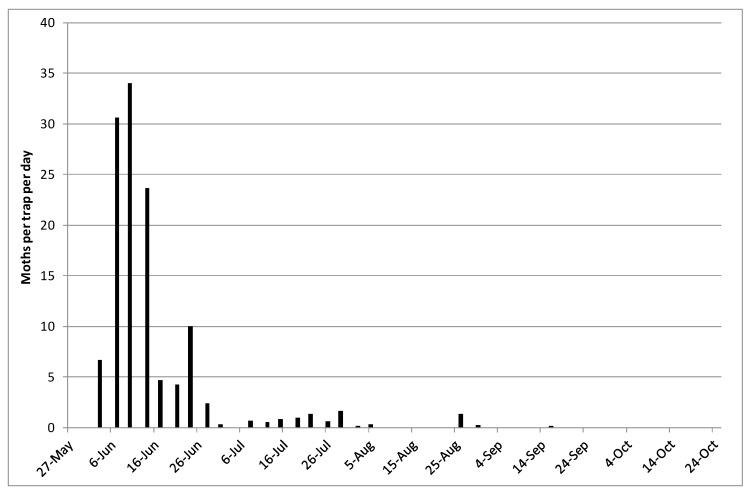
Pheromone trap captures of *P. xylostella* at Wellesbourne in 2016 using conventional Delta traps (moths per trap per day).

The Trapview system worked well and all those involved in the network were able to view the traps remotely. Very low numbers of *P. xylostella* were captured in the Trapview traps in 2015, and low numbers were also captured in the conventional pheromone traps used. In 2016, larger numbers of *P. xylostella* were captured overall by the Trapview traps (Figure 2). The single trap in Lincolnshire and one of the two traps in Kent caught the largest numbers during the period of high immigration and peak numbers of moths were captured in early June. There was evidence of a subsequent generation in Lincolnshire (first moth captured on 17 July), in the south-west (first moth captured on 10 July), and at Wellesbourne (conventional Delta traps) (Figure 3).

Although the intention had been to compare conventional traps with the Trapview traps, only one grower did this consistently. Consequently, there were three comparisons (Table 3). More than 10 times as many moths were captured by the conventional traps as by the Trapview traps.

### 3.2. Information from Databases and Twitter

Table 4 summarises the sightings of *P. xylostella* recorded on publicly available citizen science databases and Twitter (UK only) across Northern Europe in 2000–2019. Much of the yearly increase in numbers seen in the early years will be due to the steadily increasing popularity of recording observations on these citizen science portals, and so the lower numbers in the early years reflect the recent expansion in citizen science data rather than true population increases. However, some trends are also visible in the data from the last 10 years, such as the sharp population decrease across five countries from 2011 to 2012. After populations in northern Europe increased in 2013–2014, there was a subsequent steep decline in numbers recorded during 2015. Across all countries, the numbers of *P. xylostella* recorded in 2016 were the highest since 2000, with more than 1.2 million recorded in Belgium alone. Therefore, the numbers shown in Table 4 support the notion that 2016 saw *P. xylostella* migration to northern European at levels unprecedented over at least the previous decade. Since 2016, *P. xylostella* adults have been most abundant in 2019.

### 3.3. Relative Timing of Large Movements of Moths between Locations

**Figure 4:**
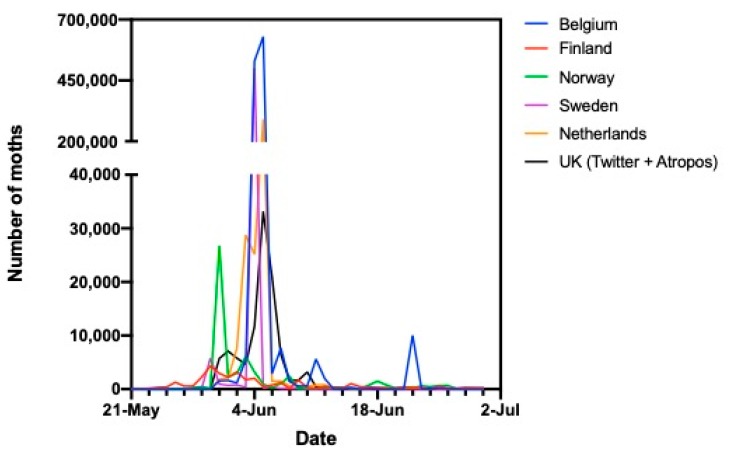
Comparison of peaks in abundance of *P. xylostella* in different European countries in 2016 (using numbers of moths seen per day by citizen scientists in each country).

**Figure 5:**
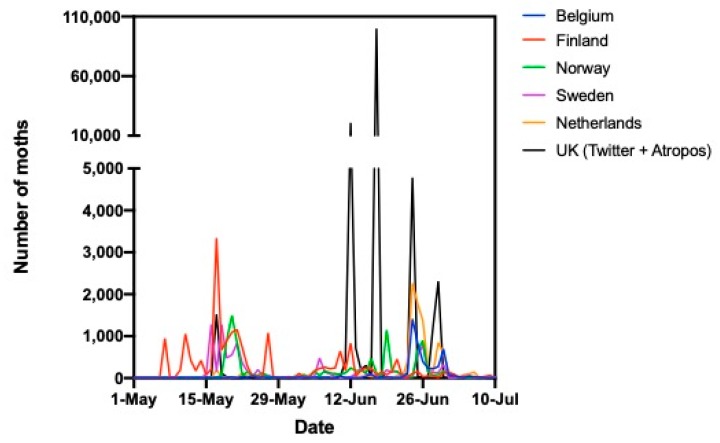
Comparison of peaks in abundance of *P. xylostella* in different European countries in 2019 (using numbers of moths seen per day by citizen scientists in each country).

Figure 4 and Figure 5 compare the timing of periods of high abundance in early summer in the different European countries in the two recent years when *P. xylostella* had been abundant. They show the numbers of moths seen per day by citizen scientists in each country. In 2016, moths were first detected in Finland, followed by Norway, and after that there were large peaks in Sweden, the UK, Belgium, and the Netherlands within three days of one another.

Again in 2019, moths were detected earliest in Finland in mid-May, then in Norway, Sweden, and the UK. Three large peaks of abundance occurred in the UK during mid to late June and the last of these coincided with peaks in Belgium and the Netherlands.

### 3.4. Predicting the Source of the Influx in 2016

**Figure 6:**
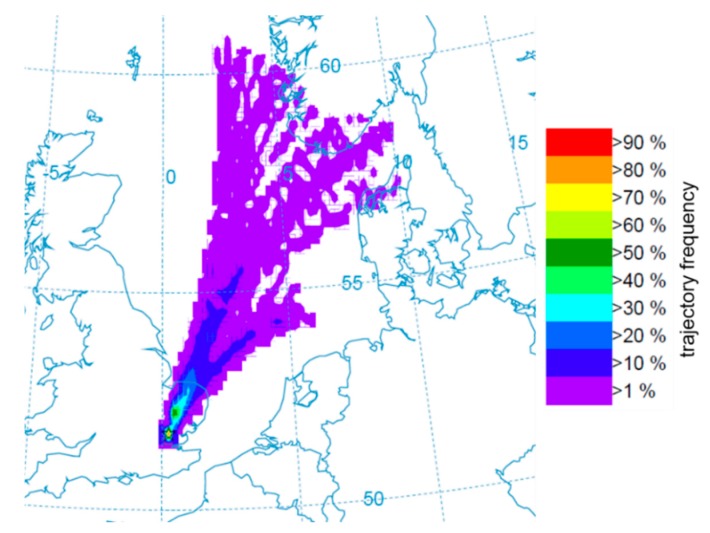
Back trajectory frequency plot for a sample record of *P. xylostella* from a light trap in Kimpton, Hertfordshire (yellow star). The colours indicate the likelihood of each 0.25° × 0.25° grid cell being the initial source location for a trajectory ending at Kimpton every 3 h from 10:00 on 30 May 2016 to 07:00 on 4 June 2016.

The resulting frequency diagram for the source location of Kimpton (51.851° N, 0.2997° W) is shown below (Figure 6). It is clear that the only trajectories that allow for movement of *P. xylostella* into the UK from overseas are those originating over land areas (unless the maximum flight duration or another flight capability of *P. xylostella* has been significantly underestimated). This suggests that the initial incursion into the UK originated from the Norwegian and Danish coastlines. This is somewhat surprising, given that there were significant numbers of *P. xylostella* in Belgium and the Netherlands during this whole period, following a build-up of populations there from mid-May onwards, and the populations there would have a much shorter distance to cover in order to arrive at the UK coastline.

**Figure 7:**
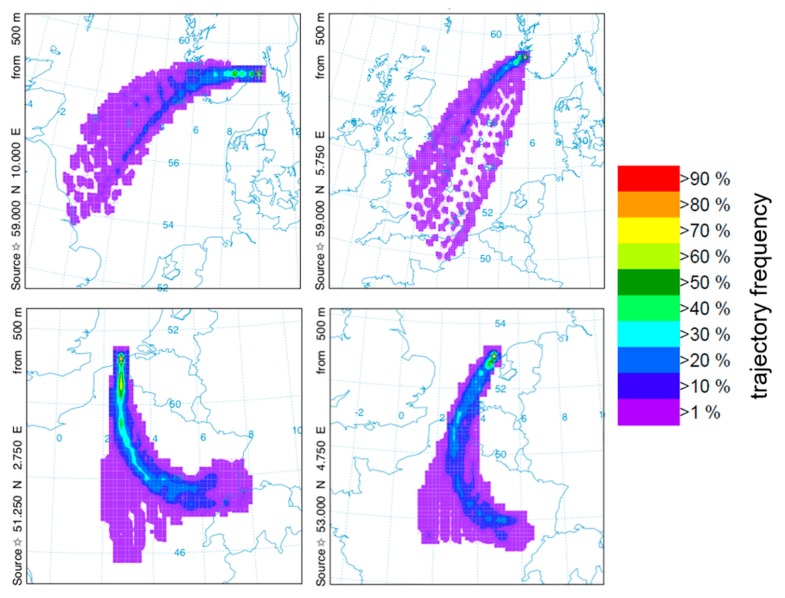
Forward trajectories from four starting locations in Norway (top left; source location 58.968° N, 9.905° E; top right; source location 59.002° N, 5.719° E), Belgium (lower left; source location 51.185° N, 2.82° E), and the Netherlands (lower right; source location 53.088° N, 4.795° E). The trajectories were started every 3 h from 19:00 on 29 May 2016 to 19:00 on 31 May 2016 and flight height was set as 500 m with a 24-h flight duration.

Given the surprising nature of this result, a series of corresponding forward trajectory models were run, initialized by citizen science records reported in Belgium, the Netherlands, and Norway during the week preceding 1 June. The model parameters were kept the same as in the backward trajectory frequency plot described above and shown in Figure 7, but these trajectories were initialized in the source locations described in the figure caption and allowed to run forwards in time for 24 h (rather than backwards as before). Thus, each coloured point in Figure 7 represents the likelihood of a particle starting in the source location ending up within a given 0.25° × 0.25° grid cell. Figure 7 shows further evidence that migration between the Norwegian coastline and eastern and southern UK was possible with a 24-h flight duration. The forward trajectories with source locations in Belgium and the Netherlands (the lower two panels in Figure 7) do not support movement into the UK from these regions due to the prevailing wind direction at that time providing support for movement in a southerly and south-easterly direction towards central France.

### 3.5. Grower Experience of the Influx of P. xylostella into the UK in 2016

Discussion at the workshop in January 2017 indicated damage levels in 2016 due to feeding by larvae of *P. xylostella* ranging from 0% to 100%, with Brussels sprout crops being some of the most affected (AHDB, 2017) [15]. Of the 13 respondents to the questionnaire, six had used pheromone traps. The large migration in 2016 was detected by growers between 31 May and 9 June and eggs were detected between late May and mid-June. Larvae were observed between late May and early July, with as many as 40 per plant.

### 3.6. Using Citizen Science and Grower Monitoring Data to Provide Warnings for UK Growers

**Figure 8:**
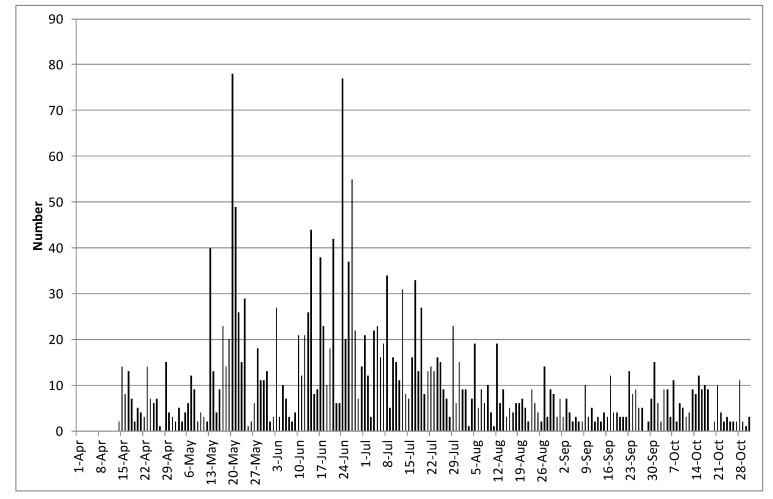
The number of visits in 2019 to the web page summarising daily information on sightings by citizen scientists in northern Europe from 1 April until 30 September.

Whilst the web pages summarising information on sightings by citizen scientists in northern Europe were updated daily, information from the network of pheromone traps arrived weekly or less frequently [16] and this was related entirely to the time the trap operators were able to commit to the activity. In 2018 and 2019 the number of visits to the web pages summarising daily information on sightings by citizen scientists in northern Europe were 1784 and 2125, respectively (from 1 April to 31 October). Over the same periods, the numbers of visits to the web pages summarising pheromone trap captures were 688 and 593, respectively. Figure 8 shows the number of visits in 2019 to the web page summarising daily information on sightings by citizen scientists in northern Europe. Email summaries were circulated to growers and advisors on a weekly basis, usually on a Monday. Large numbers of *P. xylostella* (>1000) were seen in the UK on 17 May and on 12, 17, 24, 28, and 29 June. There was a further influx (maximum 872 moths) in late July. On occasions when alerts about influxes of *P. xylostella* were provided, up to 78 individuals accessed the web page in a day. In 2019, numbers of *A. gamma* were relatively low and peak numbers were observed on 22 August.

## 4. Discussion

**Figure 9:**
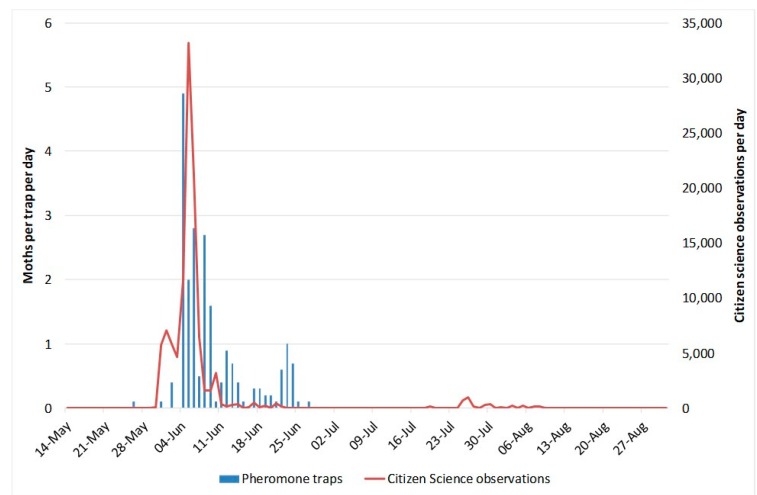
The numbers of moths captured in Trapview pheromone traps per day compared with sightings by citizen scientists per day in the UK in 2016.

The large influx of *P. xylostella* in 2016 confirmed its significance as a pest of *Brassica* crops in the UK, but it is clear that such large influxes do not occur every year. It appears, from the data presented in this paper, that *P. xylostella* was also abundant in 1996, 20 years previously, and other earlier outbreak years in the UK have been identified by Chu [9] and summarised by Zalucki and Furlong [8]. These were (since 1950) 1958, 1966, 1978, 1979, and 1980. Zalucki and Furlong do not indicate any further outbreak years between 1980 and 2008 (paper published in 2011). However, Chapman et al. [7] suggest that *P. xylostella* may also have been abundant in northern Europe in 2000 and this is partially supported by the pheromone trap captures in Lincolnshire in 2000 (Table 1). The aim of this current study was to consider information on the phenology of *P. xylostella* in the UK and to assess different ways of monitoring moth numbers to alert growers about significant influxes of moths. Large numbers of moths can arrive very suddenly and, as Figure 1 shows, eggs are laid by female moths when male moths are captured in pheromone traps. It also appears that male moths are captured in pheromone traps as soon as they arrive in the UK (Figure 9).

The two data sets that include pheromone trap captures span the periods 1996–2000 and 2006–2019, albeit at different locations. Peak numbers of moths were captured between 7 June (2016) and 17 August (1999), but when considering the two “outbreak” years (1996 and 2016), peak numbers were captured in June. Data from pheromone trap captures and plant sampling suggest that such an influx may be followed by a “second” generation that develops on crops and infestations may persist locally for longer than this. For example, an infestation on a swede crop that was visited by one of the authors (R. Collier) in mid-January 2018 had been present throughout the summer and may have been “protected” by netting covers used to exclude cabbage root fly (*Delia radicum*). It is obviously impossible to prove that all moths contributing to the second and subsequent generations originated in the UK and indeed it seems from reports on Twitter that influxes occur at almost all times of the year and that several species of migrant moth may arrive at the same time. Evidence for successful overwintering by substantial numbers of *P. xylostella* in the UK [7] is still limited. However, climate change may well alter the phenology of this species in the UK [8,10].

Data on development times of *P. xylostella* [3] showed that, for example, at a temperature of 16 °C (which might be an approximation to the mean air temperature in the summer in the UK) egg development took 6.4 days and a complete generation took approximately 33 days. The data from Liu et al. [3] were used to estimate the low temperature threshold for development by linear regression (all stages) and indicated that it was approximately 7.5 °C. From the constant temperature data provided by Liu et al. [3], between 12 and 22 °C (the range of temperatures that *P. xylostella* might be expected to experience in mid-summer in the UK), the total day-degree sum to complete a generation is about 275 day-degrees above 7.5 °C. Using weather data for 2016 from 10 locations (Cornwall in the south to Perthshire in the north) and assuming moths laid their first eggs on 1 June, then the second generation would be expected to begin in early to mid-July (range 6 July at Wellesbourne in Warwickshire to 18 July at Blairgowrie in Perthshire) [15], which was broadly in line with the information available from pheromone trap captures (Figure 2 and Figure 3). This suggests that in 2016 the second peak in moth numbers is likely to have been due to completion of a generation by the local population. Similarly, Perry [17] developed a day-degree model from data on development of *P. xylostella* that he collected in the laboratory (estimated low threshold temperature of 8.06 °C and 278 accumulated day-degrees to complete a generation). Using some of the field data shown in Table 1 he chose the starting point for day-degree accumulation as the day on which the first peak in numbers of *P. xylostella* was observed and the day by which 278 day-degrees had been accumulated was used to predict emergence of the second generation of adults. The observed and predicted dates were then compared. The minimum difference between observed and predicted dates was four days and the maximum difference was 10 days, with the mean absolute difference being 6.6 days.

The use of citizen science data for ecological and other studies is becoming more widely recognised [18,19,20]. In some cases, observations originate from individuals who have received some training in identification. If images are submitted, it is possible for experts to verify identification. In this study, there was no way of checking whether the moths had been identified correctly and the only factor that may instil confidence is that *P. xylostella* is a relatively easy species to identify, even though it is small in size. In the case of the Twitter and Atropos networks in the UK then some of the contributors are highly skilled taxonomists. They have an active network through social media and there is a certain amount of “group quality control”. When there are large influxes, sightings are also usually reported by several individuals, giving credence to the collective observations. However, there are occasions, for example, 4 and 5 June 2016 in Belgium, when one observer reported extremely high numbers of moths (500,000 on each of the two days). In addition, particularly when there are large influxes such as this, the numbers of moths reported will undoubtedly be estimates and it is certainly important to be cautious about the reliability of the data. One factor on which it is impossible to comment is “sampling intensity” since citizen scientists recording sightings of moths are not making observations over a fixed period or fixed distance—as in, for example, the RSPB Big Garden Birdwatch [21] (observations over a period of one hour) or the UK Butterfly Monitoring Scheme [22] (a protocol is used to define the walking of transects of a fixed length). This means that it is hard, for example, to compare abundance data between “countries” as there is no reliable measure of the relative sampling effort. Thus, there are certain limitations with regard to interpreting the data. However, it is clear that the citizen science data provides a very good and timely indication of large influxes of *P. xylostella* and perhaps, on some occasions, an early warning for growers in the UK when large influxes occur first in continental Europe. Obviously, the information from citizen scientists does not relate to particular crops and, for the reasons described above, it is impossible to determine how the moths are distributed throughout the landscape. However, analysis of the sightings reported on Twitter in late May–early June 2016 show that the moths were widespread [15].

There has been some consideration of the factors which lead to mass migrations of *P. xylostella* and it has been observed that migrants have smaller bodies, larger wings, and extended adult longevity compared with non-migratory forms [2]. In a laboratory study, where moths with these characteristics were produced by rearing larvae on mature plants, Campos et al. [23] suggested that “*the lesser nutritional quality and the short temporal persistence of mature plants are selective forces favoring individuals that are better prepared to abandon their habitat soon after emergence*”. In their study, and at a temperature of 25 °C, females reared on young plants laid 90% of their eggs up to the third day after emergence, whilst those that fed on new leaves from mature plants took seven days to lay 90% of their eggs. In an earlier study, Hillyer and Thorsteinson [24] showed that female *P. xylostella* were sexually mature at emergence only when kept at low larval density and provided with high quality food sources. Later, Pivnick et al. [25] investigated the effects of exposure to host plants and the density of adults on the onset of reproductive activities in *P. xylostella*. Their results indicated that there was a delay in sexual maturation in both male and female moths when the moths were kept in crowded conditions or when they were denied access to host plants. They suggested that the effects observed in their study and that of Hillyer and Thorsteinson may be an adaptation to facilitate migration under sub-optimal conditions.

It is impossible, in this study, to determine for how long moths arriving in the UK have deferred oviposition, but what does seem clear is that oviposition can begin soon after the moths arrive. Furlong at al. [2] concluded that although more is now understood about *P. xylostella*, the characteristics of migrating moths, the cues that promote migration, and whether populations make return migrations (as does *A. gamma* from the UK [26]) remain unclear. In the current study it appeared that the moths that arrived in the UK in 2016 originated from Scandinavia (and probably further east) and this appears to be similar to the origin of influxes into the UK in 1958 (June–July) and 1966 (June) described by Chu [9] and Chapman et al. [7], where back-tracks indicated that the moths had originated from countries bordering the eastern Baltic Sea. In Chapman et al.’s own study, the source of the moths in 2000 was probably the Netherlands and neighbouring countries. However, all of these insects (or the previous generation(s)) must have overwintered in warmer locations than these. It has been suggested that the moths that arrived from Finland in 1958 and 1966 might have originated in the steppes of southern Russia and that those that arrived from the Netherlands in 2000 might have overwintered in southern Europe [7].

The overall aim of this study was to find the best source of information for growers about the timing of influxes of *P. xylostella*. At present, in terms of a general warning it appears that use of citizen science data may be the best approach for timeliness. Whilst pheromone traps may give crop-specific information, the traps require very regular attention to equal the timeliness of the citizen science data. For example, whilst the web pages summarising information on sightings by citizen scientists in northern Europe were updated daily, information from the network of pheromone traps arrived weekly or less frequently and this was related entirely to the time the trap operators were able to commit to the activity [16]. Furthermore, whilst the Trapview network of traps showed promise in terms of timely access to information, the relatively limited data set described here suggests that they have low efficacy at present and may not reflect the scale of large influxes. It is obviously possible for the company to address this aspect in future, or for others to develop similar and more effective systems. Records of the numbers of visits to the web page on the University of Warwick website (Figure 8) indicated that the information was being accessed regularly and web page visits increased when growers were reminded with emails from the AHDB; there was usually a “peak” following the regular weekly pest update on a Monday. The maximum number of hits in a day was 78 and, whilst this might seem quite a low number, since the UK *Brassica* Growers Association has a membership of 105 grower businesses [27], the implication is that when warnings of large influxes were given, further information was accessed by a considerable proportion of those businesses. This information may then be used by growers to prioritise crop walking or visit pheromone traps in susceptible crops at more frequent intervals.

The modelling of trajectories (Figure 6 and Figure 7) and predictions of the arrival of migrants by the “moth community” on Twitter indicate that it may be possible to predict in advance when migrant moths are likely to arrive. This depends on two factors: (1) use of forecast weather data (particularly information about wind direction and speed) to predict the likely sources of migrant moths and (2) knowledge about the abundance of the species of interest in those locations. The former can be achieved relatively easily, whilst the latter is much harder, particularly because, at the moment, there are large gaps with regard to sources of citizen science, or indeed other, data. This includes France, Spain, Portugal, North Africa, and eastern Europe. It seems likely that very favourable conditions would be essential for the development of such large populations of insects and these would include relatively high temperatures and a good source of brassicaceous host plants.

## 5. Conclusions

The study has confirmed that *P. xylostella* is a sporadic but economically important pest of *Brassica* crops in the UK and that very large numbers of moths can arrive suddenly; their immediate sources being countries in the western part of continental Europe. Migrations most often occur in early summer but there is still insufficient information to determine where these moths or their predecessors have spent the winter. Once they have arrived, female *P. xylostella* lay eggs rapidly and since their life-cycle is relatively short it is important for *Brassica* growers to be warned rapidly. Whilst the numbers of male moths can be monitored using pheromone traps, a considerable amount of effort is required to visit these daily. A network of pheromone traps, each containing a small camera sending images to a website, is one way of obtaining information remotely, but the particular system tested in this study did not appear to be very sensitive in terms of the numbers of moths captured, although there is scope for improvement. However, *P. xylostella* is one of the species recorded by citizen scientists in several countries in northern Europe and this information is freely available through websites or Twitter. An initiative to summarise this information on a daily basis for presentation on an open web page has shown that the information has been accessed regularly and that web page visits increased when growers were reminded with emails.

## Figures and Tables

**Table 1:** Summary data from five locations in Lincolnshire, UK, in 1996–2000. Means and standard deviations (SD) are shown.

Parameters Analysed		1996	1997	1998	1999	2000
Number of sites		5	5	5	5	3
Total number moths caught	Mean	1382	225	289	37	522
	SD	321	99	226	35	67
Peak number of moths	Mean	224	49	47	14	113
	SD	25	20	41	12	24
Peak number of larvae	Mean	236	18	126	7	481
	SD	172	12	108	5	318
Date of peak number of moths	Mean	27 June	14 August	9 July	17 August	9 August
	SD	31	2	20	20	12
Date of peak number of larvae	Mean	3 July	28 August	26 July	11 August	18 August
	SD	2	3	18	25	4

**Table 2:** Captures of male *P. xylostella* in two pheromone traps at Wellesbourne in Warwickshire 2006–2019.

	Total Number of Moths Captured June–September Inclusive (2 Traps)	Peak Number of Moths	Dates When Peak Numbers Captured
2006	70	10	13 June, 25 July
2007	106	34	12 June
2008	35	6	27 June
2009	182	43	7 July
2010	36	5	15 June, 18 June
2011	75	15	5 July
2012	22	8	12 June
2013	9	3	8 August
2014	2	1	-
2015	30	10	12 June
2016	879	245	7 June
2017	41	14	7 July
2018	93	11	3 July, 10 July
2019	77	15	14 June

**Table 3:** Comparison of captures by Delta and Trapview traps in 2016.

Location	Trapping Period	Total Number Caught in Delta Trap	Total Number Caught in Trapview Trap	Ratio (Numbers in Delta Trap/Trapview Trap)
Whitnage, Devon	8 June–12 September	250	15	16.7
Preston Bowyer, Somerset	8 June–29 September	278	15	18.5
Wellesbourne, Warwickshire	1 June–30 September	879 (2 traps)	35	12.6

**Table 4:** Numbers of *P. xylostella* recorded on publicly available citizen science databases and Twitter across Northern Europe 2000–2019.

Year	Norway	Sweden	Finland	Belgium	Netherlands	UK Twitter	UK Atropos
2000	7	9	1241	89	0		
2001	1	30	584	11	0		
2002	6	27	328	47	0		
2003	1	2	576	50	13		
2004	2	4	109	18	1		
2005	0	5	629	16	0		
2006	0	13	355	243	38		
2007	1	166	5325	112	75		
2008	5	22	102	114	145		
2009	679	1594	4820	11,530	3342		
2010	205	1732	8827	1137	1478		
2011	39	178	668	236	333		
2012	74	278	3004	1136	776		
2013	2406	5610	29,429	1615	2719		
2014	4574	5068	17,116	7793	10,800		
2015	416	504	3113	1082	1186	278	642
2016	57,588	512,849	41,185	1,204,964	371,830	46,412	71,833
2017	158	113	268	1248	1099	665	354
2018	2748	1460	6056	6460	8529	511	2177
2019	12,154	10,731	19,762	9831	14,485	134,304	2875
